# Quality Control Strategies for Pit Mud Based on the Synergistic Effects of Flavor, Microorganisms and Physicochemical Factors

**DOI:** 10.3390/foods14193326

**Published:** 2025-09-25

**Authors:** Linjia Sun, Xiaofeng Zhang, Xuesi Li, Zhenhua Cao, Ming Hui, Chunmei Pan

**Affiliations:** 1 College of Biological Engineering, Henan University of Technology, Zhengzhou 450001, China; 15093068949@163.com (L.S.); huiming@haut.edu.cn (M.H.); 2 College of Food and Biological Engineering (Liquor College), Henan University of Animal Husbandry and Economy, Zhengzhou 450046, China; zhangxiaof78@126.com (X.Z.); shjykyzx@163.com (X.L.); 3 Henan Songhe Liquor Industry Co., Ltd., Zhoukou 477262, China; 15703881375@163.com

**Keywords:** pit mud microecology, functional microbial community, physicochemical properties, artificial pit mud

## Abstract

As the core of the solid-state fermentation system, the quality of pit mud is directly determined by the synergistic effects of volatile flavor compounds, microbial communities, and abiotic physicochemical factors. However, pit mud lacks systematic knowledge, especially concerning the dynamic association mechanism and threshold effect of its three components. This results in blind spots within the pit mud metabolism regulation network, which has become a bottleneck in precise pit mud quality regulation. Focusing on the volatile flavor compounds of pit mud is crucial to exploring their association with the core microbial community and physicochemical factors of pit mud, in order to cultivate high-quality pit mud. Although a large number of studies have revealed the formation mechanism of high-quality pit mud based on the three factors and cultivated artificial pit mud through microbial enhancement or synthetic flora to improve the quality. However, anaerobic fermentation is a complex system, and the complexity and dynamics of microorganisms make it difficult for biofortification and synthetic flora to effectively regulate the quality of pit mud. Therefore, this paper reviews the research progress on flavor compounds, microbial communities and abiotic factors associated with pit mud quality, deepens the understanding of their influence on pit mud quality, and proposes a precise environmental control strategy to alter the composition and content of the microbial community in the pit mud. The key to this scheme lies in constructing a correlation network through multi-omics integration to identify the physical and chemical factors related to the quality of the pit mud. During the fermentation process, intervention measures are taken on environmental parameters, ultimately effectively changing the physical and chemical factors, thereby achieving the assumption of precise control of the quality of the pit mud. This provides necessary references and inspirations for improving the quality of pit mud, cultivating artificial cellar mud, and enhancing the quality of Baijiu.

## 1. Introduction

Strong-flavor baijiu (SFB), as one of the traditional aromas of Chinese Baijiu, is distinguished by its intense aroma, mellow palate, and sweet-clean finish, securing broad consumer appeal [1]. According to the National Bureau of Statistics of China, SFB holds a 60% market share, dominating the Baijiu market [2]. Its production employs mixed grains (sorghum, corn, barley) as substrates, medium-high-temperature, Daqu as a fermentation inoculant, and pit mud as the vessel for solid-state fermentation (SSF), followed by distillation, aging, and blendin. The walls and bottoms of the fermentation pits are coated with mud, serving as the functional carrier for these pits. The fermentation that occurs within the pit mud is a distinctive feature of the SFB brewing process [3]. The pit mud matrix hosts a diverse array of syntrophic microbial consortia, whose esterase systems catalyze the production of key volatile flavor compounds. These metabolites are translocated into fermenting grains through diffusive exchange and then distilled into the spirit, thereby forming SFB’s organoleptic profile. Consequently, the quality of pit mud critically determines the sensory and chemical attributes of SFB.

**Figure 1:**
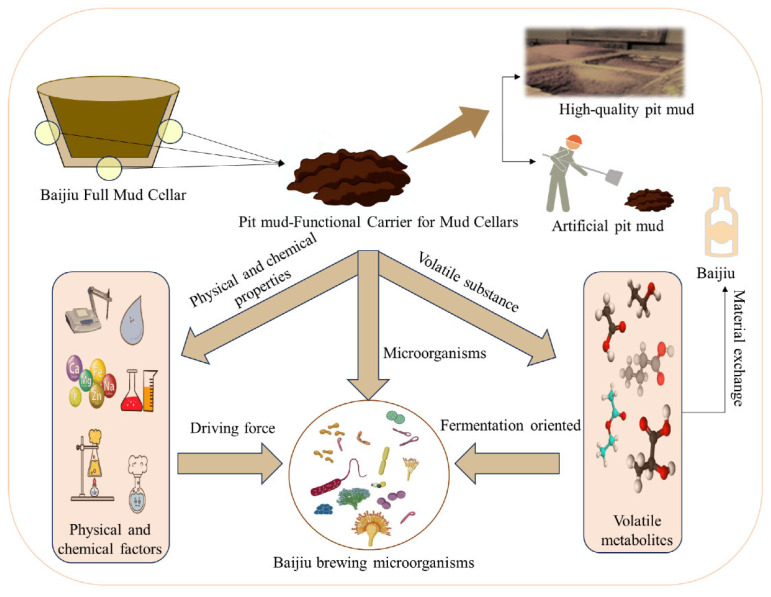
Functional carrier of the cellar mud-pit mud.

The functional properties of pit mud arise from the synergistic interplay among three key elements: volatile flavor compound profiles, microecological community structures, and physicochemical matrix parameters [4]. Premium pit mud is characterized by its dark-brown color, fine texture, and distinct cellar aroma. It contains dominant volatile compounds such as ethyl caproate, ethyl acetate, n-hexanol, and n-propanol. Conversely, degraded pit mud exhibits chromatic aberrations, coarse granularity, and malodorous notes (rancidity, raw-earth, putrescence), where indole, 3-methylindole, fluorene, and 4-methylphenol constitute the primary “mud-odor” components [4]. These volatile metabolites originate from the core pit microbiota, including *Clostridium kluyveri* (caproate-producing bacteria), lactobacilli, and methanogens, through glycolytic, fatty acid biosynthetic, and other essential metabolic pathways [5]. Meanwhile, microbial proliferation is regulated by physicochemical determinants (moisture content, ammonium nitrogen, and humic substances) within the pit ecosystem [6]. Therefore, analyzing the correlation between volatile flavor compounds, microbial communities, and the physical and chemical properties of pit mud is of significant guiding importance for enhancing both the quality of pit mud and the quality of Baijiu (Figure 1).

Pit mud quality degrades over time due to microbial imbalances and physicochemical changes. Pit mud is fraught with uncertainty and complexity during its extended period of use [7,8,9]. Artificial pit mud (APM) is cultivated through modern biotechnology by simulating the ecological characteristics of traditional matured pit mud. Nutrients, high-quality pit mud and composite functional bacteria (e.g., pure strains of capric acid bacteria) are added to the pit mud soil to rapidly cultivate functionalized pit mud with high-quality cellar fermentation characteristics [10,11,12]. This process enhances the content of key flavor substances in pit mud within a short period, achieving the goal of improving pit mud quality [13,14]. Modern studies have analyzed the bacterial structure of APM by Macrogenomics/Macrotranscriptomics, constructed Core functional guilds (e.g., syntrophic Clostridium-methanogen systems) for direct inoculation, and constructed Synthetic Microbial Communities (SynComs) for precision-fermented APM cultivation. These approaches enhance pit mud stability and distillate production efficiency through ecological modulation [15,16,17].

**Figure 2:**
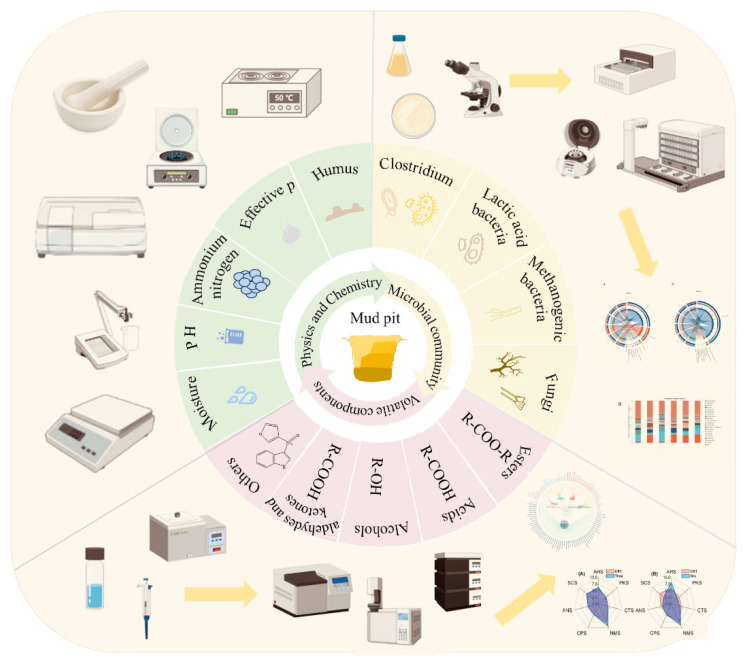
Three major factors affecting the quality of pit mud.

Recent advances in analytical biotechnology, particularly in gas chromatography-mass spectrometry (GC-MS), gas chromatography-olfactometry (GC-O), and meta-omics platforms (such as metagenomics and metatranscriptomics), have enabled comprehensive profiling of volatile flavor compounds in pit mud and the decoding of microbial consortia structure and function [8,18]. The volatile flavor compounds, microbial communities, and physicochemical properties of pit mud (Figure 2) are crucial for maintaining functional homeostasis and ensuring precise flavor regulation. This paper reviews the research status of volatile flavor compounds, main microbial communities, and physicochemical properties in pit mud, as well as their roles in the pit mud cultivation process, are reviewed. The interaction mechanism among these three factors is analyzed, and the potential development direction of APM cultivation in the future, along with the challenges that may be encountered, are discussed. Aims to provide references for APM cultivation, quality control, and the improvement of SFB quality.

## 2. Composition and Origins of Volatile Flavor Compounds, Microbial Communities, and Physicochemical Factors in Pit Mud

### 2.1. Volatile Flavor Compound Composition and Origins

**Figure 3:**
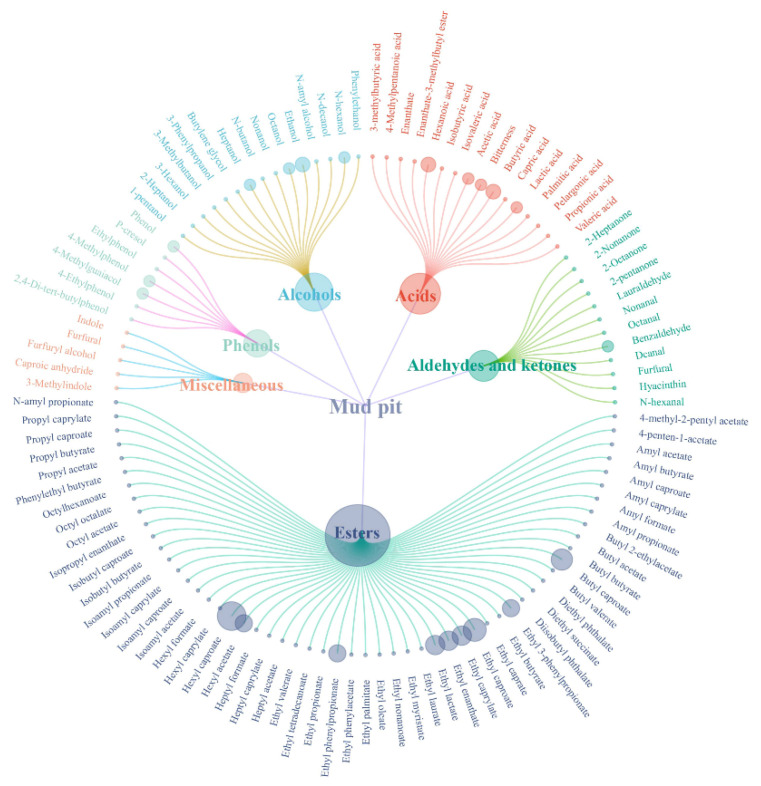
Composition of volatile flavor compounds in pit mud.

The composition of volatile flavor compounds in pit mud serves as a sensory indicator of its quality. Pit mud contains hundreds of structurally diverse volatile compounds, predominantly esters, acids, alcohols, aldehydes, and ketones (Figure 3). These metabolites originate primarily from microbial metabolic activity within the pit ecosystem [19].

Esters are the main components of the volatile flavor compounds in the pit mud, with strong fruity, floral and sweet odors, mainly including ethyl caproate (400–7607 mg/L), ethyl acetate (about 25.4–1773 mg/L in SFB), ethyl lactate (about 4.40–1067 mg/L in SFB), ethyl butyrate (about 45.0–622 mg/L), etc., which accounted for about 44.72–68.38% of the total volatile substances in the pit mud [20,21,22]. Headspace SPME-GC-MS analyses reveal significantly higher ester concentrations in aged premium pit mud (71.6–74.4%) versus new pit mud (37.3–53.1%) and degraded pit mud [23]. By using GC-MS to compare esters in different qualities of pit mud, it was found that the content of esters in poor-quality pit mud was significantly lower than that in normal pit mud [4]. Ester biosynthesis occurs via two primary pathways, one is the transesterification reaction of microorganisms such as yeast, and the other is the esterification reaction of acids (e.g., hexanoic acid, butyric acid) and alcohols (mainly ethanol, produced by functional microorganisms, such as brewer’s yeast) [24]. During the cultivation of pit mud, the ester content can be significantly increased by adding caproate-producing bacteria to the mud [25].

Acids are the secondary volatile fraction in pit mud, which serve not only as precursors for ester production, but also as significant contributors to its aroma and flavor. These primarily include hexanoic acid, caprylic acid, acetic acid, butyric acid and other volatile organic acids, whose content (20.54–46.63%) is second only to esters in the volatile flavor compounds in pit mud [19]. The content of organic acids in pit mud increases as the cellar ages, and the distribution of organic acids varies in pit mud of different qualities, with high-quality pit mud having a higher content of organic acids [26]. The common synthesis pathway for organic acids involves the elongation of the carboxylate chain, utilizing ethanol and lactic acid as electron donors. It has been observed that pyruvic acid is the key substrate for the formation of organic acids in pit mud. Consequently, the metabolism of pyruvic acid and butyric acid may be the crucial metabolic pathways responsible for the varying content of organic acids in pit mud of differing qualities [27].

Alcohols in pit mud (4.68–14.29%) are important aroma compounds, which can generate esters through esterification with acids in pit mud [28]. Alcohols have a low boiling point and are easy to volatilize. During this process, they can “drag and drop” other components to volatilize, which can better support the ester aroma in the pit mud. This aroma is mainly dominated by ethanol and hexanol, butanol and other Fusel alcohols (monohydric alcohols with more than three carbons) [29]. The content of alcohols in high-quality pit mud is lower than that in new pit mud, and the content of alcohols in poor-quality pit mud is lower than that in normal pit mud [4]. Ethanol in pit mud is produced from glucose through the glycolysis pathway to generate pyruvic acid, and then further processed through the tricarboxylic acid cycle to generate α-keto acid, followed by the conversion of α-keto acids into ethanol. Meanwhile, higher alcohols, such as hexanol and butanol, are generated by yeast under abnormal metabolic conditions utilizing the decarboxylation and deamination of amino acids from the environment (e.g., isoamyl alcohol is generated from leucine) [30]. Butanol in pit mud is synthesized by butanol-producing bacteria (e.g., *Caproiciproducens*) via the glycometabolic synthesis pathway (Harris pathway), and the butanol content in the pit mud is determined by the quantity of butanol-producing bacteria in the pit mud [31].

Aldehydes and ketones (0.39–1.56%) are important components of volatile flavor compounds in pit mud, and common aldehydes and ketones in pit mud include acetaldehyde, benzaldehyde, 2,3-butanedione, 3-hydroxy-2-butanone, and so on [32]. Aldehydes usually have a pungent odor, while ketones are not inherently flavor substances. However, small amounts of short-chain carbonyl compounds exhibit caramel odor and fruity sweetness, which play the role of buffering and balancing in the aroma of pit mud [33]. The sensory thresholds of aldehydes and ketones in an alcohol-water solution were 16.51–466,321.08 μg/L [19]. The content of aldehydes (1.17%) was higher in normal pit mud, whereas the ketone content was higher in poorer quality pit mud than in normal ones [4]. The formation of aldehyde-ketone substances originates from the Strecker degradation of amino acids and the synthesis process of glucose. The Strecker degradation of amino acids produces phenylethanal and isopentanal, while glucose and amino acids are directly synthesized by microorganisms to generate acetaldehyde. Ketone substances are produced through the β-oxidation pathway of fatty acids, and acetone, 2,3-butanediol, and 2,3-butanedione can directly convert into each other [34].

During the fermentation process, the volatile substances in the pit mud encompass not only esters, acids, alcohols, aldehydes, and ketones but also phenols, terpenes, pyrazines, among others [35]. Analysis and comparison of different quality pit mud using detection techniques such as GC-O-MS and GC-MS have revealed that 3-methylindole produced by degradation of tryptophan, and 4-methylphenol synthesized by Clostridium perfringens, have a strong malodor and are the main sources of pit mud odor [4,36]. Reducing their contents during the pit mud cultivation process can significantly improve the quality of the pit mud. In addition, phenolics in pit mud also pose a threat to the quality of pit mud [37]. Currently, there are relatively few studies on the formation mechanism and inhibition of off-flavor substances in pit mud. Therefore, it is of great significance to investigate in depth the sources and compositions of volatile flavor compounds in pit mud during the fermentation process to improve the quality of pit mud.

The flavor compounds such as caproate and butyrate in pit mud are produced by microbial metabolism, but microorganisms can also produce flavor compounds such as p-cresol and phenol. It has been proven feasible to use microbial reduction strategies to effectively obtain functional microbial communities that produce caproate while avoiding the production of flavor compounds [38]. However, using such method requires a profound comprehension of the core microbial community in the pit mud.

### 2.2. Composition and Origin of the Microbial Community in the Pit Mud

Pit mud represents a complex prokaryote-dominated microbial ecosystem (comprising bacteria and archaea), with minor eukaryotic constituents. Suboptimal pit mud quality inhibits proliferation of key brewing and aroma producing microorganisms [5]. As a microbial reservoir central to fermentation, pit mud develops an intricate community structure through prolonged domestication. High-quality pit mud exhibits a stable consortium dominated by caproic acid-producing bacteria, methanogenic archaea, and fermentative yeasts (Table 1), which originate from diverse environmental reservoirs. These microorganisms, originating from soil, air, and raw materials, gradually colonize during the early stages of pit construction [39]. Furthermore, microorganisms associated with brewing substrates and starter cultures progressively transfer into the pit mud during fermentation, further enhancing the microbial diversity [40]. Through prolonged use under SSF conditions, cyclical fermentation processes, and periodic pit sealing, the pit mud microbiota undergoes continuous ecological succession, ultimately establishing a stable, complex microbial ecosystem.

Caproate-producing bacteria are typical functional bacteria found in old pit mud, dominating the bacterial community in both cellar age and spatial location. The common caproic acid-producing bacteria in pit mud primarily belong to the genera *Clostridium*, *Capnobacterium* spp., and *Hexobacterium* [55,56]. These bacteria exhibit a robust fermentative capacity, utilizing substrates such as sugars, fatty acids, alcohols, and sulfates to synthesize short- and medium-chain fatty acids (C4–C6) through the reverse β-oxidation (r-BOX) pathway. This metabolic activity is crucial for the development of pit mud aroma, with high-quality pit mud showing a significantly greater relative abundance of caproate producers compared to other genera [57]. The application of caproate bacteria (such as *Rummeliibacillus suwonensis* DFN-1) in pit mud can significantly improve the quality of the pit mud [14]. Co-culture of Caproate-producing bacteria with other microorganisms, such as *Syntrophococcus*, can significantly increase the content of acidic substances such as butyric acid, caproic acid and valeric acid in the pit mud [58,59]. The abundance of caproate-producing taxa progressively increases with pit age, driving the biogeochemical cycling of carbon, nitrogen, and sulfur, and reinforcing microbial structural stability through population-level interactions [48]. The isolation of pure strains is essential for studying the metabolic characteristics of Caproate-producing bacteria. However, due to the limitations of current culture methods for these bacteria, a large number of Caproate-producing bacteria in the pit mud remain unisolated. In the process of isolating Caproate-producing bacteria, more innovative and advanced techniques, such as flow cytometry and microfluidic sorting systems, could be considered. These methods, when combined with bioinformatics technology, could help explore the resources of Caproate-producing bacteria.

Lactic acid bacteria are the dominant bacterial genera and biomarkers in nascent pit mud ecosystems, including species such as *Lactobacillus* spp., *Lactococcus* spp. and *Sclerococcus* spp. [60]. Amplicon-based analyses indicate that the abundance of lactic acid bacteria exhibits an inverse correlation with the age of the pit [61]. During the aging process of pit mud, lactic acid bacteria can play a variety of roles: firstly, they generate volatile flavor compounds such as lactic acid, ethyl lactate, acetic acid, and ethyl acetate and other aromatic substances [62]; Secondly, to provide nutrition for the pit mud microorganisms, lactic acid bacteria increase the levels of amino acids, vitamins, and other nutrients in the grains through metabolic activity, thereby promoting the growth and reproduction of microorganisms such as caproate-producing bacteria [63]. Third, the maintenance of the pit mud microecological environment is crucial. The metabolism of Lactobacillus produces lactic acid, which helps to maintain the pH of the pit mud, inhibit the growth of unwanted bacteria, and stabilize its quality [64]. Currently, research into lactic acid bacteria within pit mud remains in an exploratory phase and has not yet fully identified or recognized all lactic acid bacteria species. As research into these bacteria progresses, it will become more profound, which will provide favorable support for the development of APM and the quality control of pit mud.

Methanogenic bacteria are more active in their metabolism at the end of anaerobic fermentation. Some of their metabolites, such as acetic acid, are significant flavor components of the pit mud. The methanogenic bacteria found in the pit mud mainly include *Methanobrevibacter*, *Methanobacterium* and *Methanoculleus* [65]. Fatty acid degradation constitutes the rate-limiting step in the anaerobic digestion process of pit mud. When fatty acid accumulation occurs, it disrupts the balance of the anaerobic digestion reaction. Methanogenic bacteria are a potent solution to the issue of fatty acid accumulation, effectively utilizing hydrogen produced by fermentative bacteria (such as Caproate-producing bacteria) and converting it into methane. This process prevents hydrogen accumulation during fermentation, thereby inhibiting the degradation of fatty acids [5]. Methanogens interact with *Clostridium* sp. *nov* and other clostridial genera. It has been found that the consumption of hydrogen produced during hexanoic acid synthesis by hydrogenotrophic methanogens promotes the synthesis of hexanoic acid through the process of interspecies hydrogen transfer [66]. The study of methanogens in pit mud faces many challenges, because methanogens are mostly strict anaerobes, demanding growth conditions, and relatively slow growth, and at the same time the microbial community in the pit mud is complex, methanogens and other microorganisms have symbiotic or competitive relationships, and their isolation and cultivation is difficult to obtain pure cultures [67].

To date, studies on the microorganisms in pit mud have primarily focused on prokaryotic organisms such as bacteria and archaea, and fewer studies have been conducted on fungi in pit mud [68]. Although the abundance of fungi in pit mud is small, some studies have shown that fungi play an important role in cellar aging [69]. *Penicillium* spp. and *Aspergillus* spp. are predominant fungal genera, exhibiting robust saccharification capabilities. They produce a large number of saccharification enzymes during Baijiu fermentation, degrading starch in the raw material into reducing sugars, which can be directly utilized by yeasts [70]. The fungal content varies among different types of pit mud, and the pit wall mud exhibits greater richness and diversity in fungal species compared to the pit bottom mud. This may be because the vast majority of actinomycetes are aerobic or facultative anaerobic fungi, which are suitable for growth and reproduction in the upper layer of pit mud with a large contact area with oxygen and high oxygen content [71].

With the development of microbial detection technology, the understanding of microorganisms in the pit mud has become increasingly profound. However, the pit mud microorganisms are diverse, with most being anaerobic bacteria or extremophiles. and it is challenging to obtain their effective pure cultures using existing microbial isolation methods, which significantly impedes the in-depth study of pit mud microorganisms and the cultivation of the pit mud [72]. To overcome this barrier, researchers have begun to investigate new culturing strategies based on the physical and chemical properties of the pit mud. Analyzing these characteristics can more accurately simulate the microecological environment of the pit mud, providing a theoretical basis for the reconstruction of the microbial community, optimization of its function, and the cultivation of the pit mud.

### 2.3. Composition and Sources of Physical and Chemical Factors

The physicochemical properties of pit mud are key parameters for assessing its quality. These properties critically shape the microbial habitat within the pit mud, largely determining the structure and succession of microbial communities, and ultimately influencing the profile of volatile flavor compounds [22]. Significant variations exist in the physicochemical properties of pit mud across different quality grades. Moreover, multiple key properties-including moisture content, available phosphorus, ammonium nitrogen, and humus-exhibit substantial temporal dynamics correlated with fermentation duration [5].

Moisture is a crucial element for the normal reproduction and metabolism of microorganisms. Yellow water, bacterial culture solution, wine tails and metabolic water of microorganisms are the main sources of moisture in the pit mud [73]. The moisture content in pit mud exhibits a dynamic trend during the fermentation process: it initially increases, then decreases, and finally rises slowly. As the pit mud ages, its moisture content tends to be higher. The moisture content of high-quality pit mud ranges between 40% and 50%. Pit mud moisture with the fermentation process showed a dynamic trend of first rise, then fall, and finally rise slowly. As the pit mud ages, its moisture content tends to be higher. The moisture content of high-quality pit mud ranges between 40% and 50% [5]. A low moisture content in the pit mud can easily cause cellar sloughing and inhibit the growth of microorganisms, whereas a high moisture content can lead to difficulties in hanging the wall and potential cellar collapse [64]. The water in the pit mud is a “bridge” connecting microbial metabolism with chemical reactions. The nutrients required by microorganisms need to borrow water as a medium to be effectively absorbed and utilized [64]. During production practice, the water content in the pit mud can be adjusted through methods such as sprinkling conservation liquid and adding auxiliary materials.

PH serves as a robust indicator of the fermentation status and a key determinant of quality in pit mud ecosystems. The dynamics of the pit mud pH are primarily governed by microbial metabolism, organic matter decomposition, and the addition of exogenous substances [47]. During fermentation, a lower pH favors the enrichment of acid-tolerant/acidophilic bacteria (e.g., lactic acid bacteria), which elevates lactic acid and related acid concentrations. Conversely, a higher pH may promote the proliferation of ester-synthesizing microorganisms, thereby enhancing ester content [60]. It is widely accepted that high-quality pit mud has a pH level near neutral. The pH of the pit mud can be fine-tuned to enhance its quality by incorporating sodium hydroxide and introducing acid-producing microorganisms, such as Clostridium graminearum, during the cultivation process [74].

Ammonium nitrogen content serves as a bioindicator for the maturation of pit mud, primarily generated through the microbial decomposition of organic matter and stabilized in ionic/salt forms [75,76]. Significant heterogeneity in ammonium nitrogen levels exists across different grades of pit mud quality (ranging from 50 to 350 mg/100 g dry weight). High-quality specimens exhibit markedly elevated concentrations compared to new pit mud, which in turn surpass those in deteriorated pit mud [77]. During the use of pit mud, the ammonium nitrogen content will gradually decline, but the ammonium nitrogen content of high-quality pit mud should be maintained at a certain level [78]. Therefore, in the maintenance process of pit mud it is necessary to continuously supplement ammonium nitrogen. The appropriate amount of ammonium nitrogen is essential to maintain the stability of the pit mud and to improve the quality of SFB [75].

Effective phosphorus refers to acid-soluble phosphorus and adsorbed phosphorus that can be utilized by microorganisms. In pit mud, this form of phosphorus is produced by the decomposition of microbial cells, except that it is brought in the ingredients [77]. Phosphorus is an effective source of energy for organisms and is a primary component of biofilm and nucleic acid. It also plays a role in a variety of biological metabolic pathways, including protein synthesis, esterification of acetic acid and ethanol, etc., which is conducive to the growth and reproduction of microorganisms [79]. Phosphorus content, which typically ranges from 500 to 3500 mg/kg, is positively correlated with the microbial biomass and metabolism which can be used as an indirect indicator of biomass, and may also indirectly reflect the pit mud maturity [41]. The content of effective phosphorus increases as the age of the pit mud advances, and the increase in effective phosphorus content in the pit mud may be attributed to the transportation of water-soluble phosphorus from the lees via yellow seepage water, which is then absorbed by organisms. Additionally, the organic phosphorus is converted into effective phosphorus by the mineralization and acid decomposition [79]. Determining the content of effective phosphorus in pit mud can reflect the activity of cellar microorganisms, thereby characterizing the quality of pit mud [80].

Humus serves as a key biomarker of pit mud maturation, synthesized through microbial degradation of organic substrates in distiller’s grains, yellow seepage water, and pit wall materials. As the dominant carbon repository and nutrient reservoir (C, N, P), it provides essential micronutrients and macronutrients sustaining the pit mud microbiota [81]. Humus content demonstrates positive correlation with pit age, with premium-grade pit mud maintaining concentrations of 10–18% (*w*/*w* dry basis) [74]. Humus enhances the metabolic activity of microorganisms in the pit mud, which has a positive impact on the quality of the pit mud. Cultivating pit mud based on materials such as old pit mud and distiller’s grains can increase the content of humus in the pit mud [82].

Key physicochemical factors governing pit mud quality include pH, ammonium nitrogen concentration, and available phosphorus content. These parameters represent reliable indicators for assessing pit mud quality [35]. Incorporating these methods into routine pit mud analysis enables rapid quality determination. Compared to the traditional method of assessing pit mud quality based on microbial community composition, this physicochemical approach offers greater convenience, speed, and significantly lower detection costs. However, current understanding of the relationship between pit mud physicochemical properties and its overall quality remains insufficient. Furthermore, research exploring the modulation of pit mud microbiota via physicochemical property adjustments to control Baijiu flavor compound formation is limited. Future investigations should focus on elucidating the complex interplay between pit mud physicochemical properties and microbial fermentation environments. This knowledge will facilitate the development of comprehensive evaluation indices for pit mud quality and strategies for stabilizing it.

## 3. Correlation Analysis of Volatile Flavor Compounds, Microbial Community and Physicochemical Properties of Pit Mud

### 3.1. Correlation Between Volatile Flavor Compounds and Microbial Community Composition in Pit Mud

The composition of major volatile flavor compounds in pit mud is mainly produced by microbial metabolism, but the vague understanding of how the microbiota of pit mud affects the volatile metabolic components of pit mud has long hindered the development of pit mud quality control and APM culture [83,84]. In recent years, with the rapid development of genomics technology, researchers have utilized multi-omics techniques such as metagenomics, proteomics, and metabolomics to accurately track the changes in microbial communities and the fluctuations of metabolites during fermentation. They have also established association network analyses between volatile flavor compounds and microbial communities from multiple perspectives (such as redundancy analysis, canonical correspondence analysis), gene expression prediction, gene function enrichment, and functional enrichment analysis, and have drawn association network analysis diagrams between volatile flavor compounds and microbial communities. This laid the foundation for identifying the microbial community related to volatile flavor compounds and establishing targeted flavor control based on the microbial community for improving pit mud quality [53,66].

Research confirms that volatile flavor compounds in pit mud are strongly correlated with dominant microbial taxa (relative abundance >1%) during fermentation [85]. Among these compounds, caproic acid has the highest content. Using the Spearman correlation coefficient combined with high-throughput sequencing and GC-MS to analyze different quality cellar mud samples, it was discovered that microorganisms of the genus Clostridium species (including *Caproiciproducens* and other *Clostridium*.) can catalyze the synthesis of hexanoic acid through the acetyl-CoA pathway. Additionally, microorganisms from the genus can extend butyric acid to caproic acid via the β-oxidation. The relative abundance of these microorganisms was found to be positively correlated with the content of flavor substances such as caproic acid and ethyl caproate in the pit mud. *Clostridium* spp. are considered as one of the key microflora contributing to the synthesis of short- and medium-chain fatty acids such as caproic acid and butyric acid [83]. Whereas *Lactobacillus* produces lactic acid to inhibit esterase activity, *Bacillus* preferentially utilizes branched-chain amino acids to decrease the supply of α-ketoglutarate, and *Aspergillus* competitively depletes free fatty acids (with a 52% reduction in hexanoic acid detected by GC-MS), its relative abundance is negatively correlated with, among others, hexanoic acid [62]. *Clostridium* is not only associated with the production of acids and esters in the pit mud but also plays a role in the formation of alcohols. It has been discovered that *Clostridium* can convert palmitic acid to n-hexanol via the β-oxidation pathway. Additionally, *Methanosarcina* can facilitate ethanol oxidation through the hydrotrophic methanogenic pathway, and its relative abundance is inversely correlated with hexanoic acid, among other compounds [86]. The relative abundance of *Methanosarcina* was positively correlated with the content of ethanol and other alcohols in the pit mud. *Thermoactinomyces* may inhibit ethanol accumulation due to competition for carbon sources. *Lactobacillus* inhibits the enzyme activity related to ethanol production, and its relative abundance is negatively correlated with hexanoic acid [86].

The formation of odorous substances such as 3-methylindole, 4-methylphenol and p-cresol in pit mud is mainly closely related to the amino acid metabolism and lignin degradation process of microorganisms [87]. Metagenomic analyses confirmed that the concentration of these substances showed significant correlation with the abundance of specific microbial genera [38]. *Clostridium* promotes the conversion of aromatic amino acids to 4-methylphenol via the phenylalanine deaminase pathway; *Methanospirillaceae* influences tryptophan catabolism, possibly through cross-domain metabolic collaborations, which is significantly and positively correlated with the accumulation of 3-methylindole; and *Desulfovibrio* is involved in the sulfate. The sulfide produced by *Desulfovibrio* in the sulfate reduction process has a synergistic effect with phenolics, and their relative abundance is positively correlated with the content of odorants in the pit mud; the phenolic acid decarboxylase gene carried by *Lactobacillus* can increase the degradation rate of p-cresol by 42%, and the lignin peroxidase contained in *Actinobacteria* can convert 4-methylphenol into quinones, and its relative abundance is positively correlated with the accumulation of 3-methylindole and negatively correlated with the content of odorants in the pit mud, among others [88,89].

Pit mud microbiota modulate the flavor profile of SFB through metabolite-mediated regulation of volatile compound composition. Modern studies utilize metagenomic and metatranscriptomic data to analysis core functional consortia (such as Clostridium-methanogen syntrophy systems) for APM inoculation. This method rapidly enhances key flavor compounds such as hexanoic acid (150–220% increase within 60 days, demonstrating targeted quality improvement [13,14]. However, this microbial-based strategy for enhancing quality still encounters several challenges during practical production and application. On the one hand, it is difficult to isolate the complex microorganisms in the pit mud, and the harsh culture conditions makes it challenging to regulate the quality. On the other hand, the microorganisms are susceptible to the environmental perturbations that cause imbalance in the colony structure, which leads to unstable quality control effects [90]. The research shows that the regulation of the physicochemical factors of the pit mud may be able to solve the current stability problem of APM quality [5,91].

### 3.2. Correlation Analysis Between Microbial Community of Pit Mud and Physicochemical Factors

The structural succession and metabolic activities of the pit mud microbial community are significantly regulated by a gradient of chemotactic factors, and their population dynamics are directly related to the formation of volatile flavor substances [22,92]. Functional bacteria (mainly *Clostridium*, *Acetobacterium*, and *Syntrophococcus*) in high-quality pit mud establish competitive advantages through microenvironments with high concentrations of organic acids, whereas newly cell-identifying bacteria, such as *Bacillus*, show low metabolic adaptation [19]. Therefore, an in-depth study of the correlation between the microorganisms in pit mud and physicochemical factors, using the main genera of pit mud as the target point, can aid in further guiding the regulation of pit mud quality.

In high-quality aged pit mud, the peak relative abundance of *Clostridium* spp. was significantly influenced by ammonium nitrogen, bioavailable phosphorus, and pH. Ammonium nitrogen-the primary nitrogen source in pit mud-exhibited a linear positive correlation with the abundance of *Clostridium* spp. within the range of 120–150 mg/kg. The metabolic activity of *Clostridium* spp. increased 1.8 to 2.3 fold when bioavailable phosphorus exceeded 0.45 g/kg, while abundance declined significantly at pH > 5.6 [93]. Conversely, in new pit mud, maximal *Clostridium* spp. abundance correlated positively with pH and ammonium nitrogen [64]. A comparative analysis of pit mud quality tiers indicated that the concentrations of pH, ammonium nitrogen, and bioavailable phosphorus were significantly and positively correlated with acid-producing functional taxa (such as *Clostridium*, *Methanobacterium*, and *Synechococcus*, but were negatively correlated with Bacillus and Lactobacillus [94]. The aforementioned studies indicate that the metabolic activity and enzymatic reaction process of pit mud microorganisms can be regulated by changing the physicochemical properties of the pit mud, dynamically changing the composition and structure of the microflora of the pit mud, so as to achieve the purpose of controlling the volatile flavor substances of the pit mud.

## 4. Traditional Pit Mud Quality Control Strategy

The brewing process utilizes high-quality old pit mud in the cellar aroma, which has a special microbial community structure and relatively stable physicochemical properties. The traditional pit mud requires a long natural maturation period. During baijiu production, operational processes and other factor can lead to pit mud degradation and aging phenomenon, which diminishes the quality of pit mud and results in significant losses for baijiu production [95]. Pit mud quality control is the use of modern biotechnology, thus realizing the strengthening of pit mud quality [96]. Currently the regulation of pit mud quality mainly focuses on employing modern biotechnological methods, such as inoculating specific functional microorganisms, and constructing simplified microbial communities. These biological approaches enrich the beneficial functional flora within the pit mud, change the composition of flavor substances within it, and thereby enhance the quality of the pit mud.

**Figure 4:**
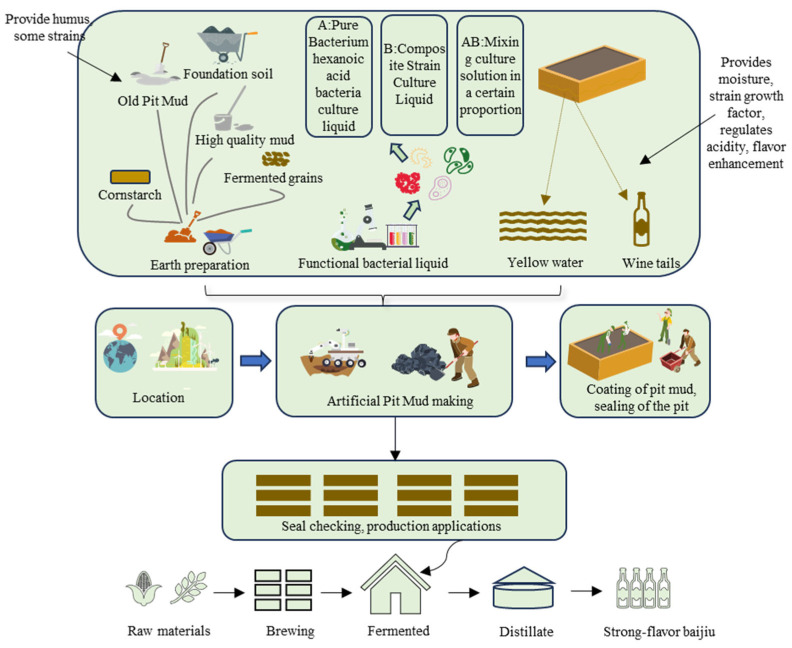
Artificial Pit Mud Production and Application.

High-quality pit mud is derived from yellow or purple-red mud, which is mixed with old pit mud, Daqu, fermented grains, and other components. A specific proportion of yellow water, liquor tails, and functional bacterial agents (such as caproic acid bacteria) are then added. After a period of cultivation, samples are taken and tested according to relevant quality standards. Once approved, the mud can be utilized for production and application [13], as illustrated in Figure 4. The addition of caproic acid-producing bacteria, such as *Clostridium rumenum* (*Clostridium* sp.), into the functional bacterial agent can reduce the population of heterobacteria in the pit mud, increase the count of beneficial bacteria, and thus cause a trend of rising accumulation of the four major esters in the pit mud. This ultimately leads to a significant improvement in the quality of the pit mud [97]. *Caproicibacterium lactatifermentans* is the primary hexanoate-producing bacterium in high-quality pit mud. Inoculating *Caproicibacterium lactatifermentans* into pit mud can result in the rapid production of abundant fatty acids and ethyl esters [98]. 225 genera of microorganisms have been detected in pit mud, but not all are beneficial for cellar flavor compounds. For instance, *Clostridium aminovalericum*, *Eubacterium contortum,* and *Clostridium fermenticellae* have been identified as p-cresol-producing bacteria and are the main cause of off-flavor odors in pit mud. The anaerobic bacteria that are the main producers of volatile flavor compounds in pit mud may be limited to just a few species [99]. Synthetic microbiomes are functionalized microbial communities created by combining pre-selected purified or enriched cultures, essentially representing strategic simplifications or engineered reconfigurations of natural communities [10]. Studies have indicated that a simplified microbial community in pit mud, which efficiently produces hexanoic acid without generating odoriferous flavor compounds, can be established using a top-down approach. This method can be applied to the Baijiu SSF (solid-state fermentation) process, significantly enhancing the flavor of Baijiu [10].

Accurately identifying functional microbial species within pit mud, revealing the structural changes in microbial communities during SSF, and elucidating the mechanisms behind community self-assembly are crucial for constructing a microbial community that produces flavor compounds without generating off-flavors, thereby significantly enhancing the quality of pit mud [100]. However, the anaerobic fermentation of pit mud during Baijiu brewing is a complex fermentation system, and most of the microorganisms in the pit mud are anaerobic microorganisms, which are difficult to be separated. This difficulty hampers the control of pit mud quality through microbial methods.

## 5. A Trinity of Regulatory Strategies Based on Physicochemical Properties

In the SSF system, the physicochemical environment of the pit mud is dynamically changing. This dynamic change can drive the growth, reproduction, and enrichment of microbial communities. Consequently, abiotic regulation that alters the physicochemical properties of the pit mud to enrich its functional microorganisms can compensate for the inadequacies of microbial regulation in controlling pit mud quality. It has become an effective measure for managing the quality of the pit mud [101]. However, relying solely on physical and chemical factors is insufficient. The changes in microbial community structure must also be considered. Microbial metabolites are essential precursors to flavor substances, and the relationships among these elements must be clearly understood. In recent years, with the advent of artificial intelligence and other technologies, the field of fermentation has undergone significant transformation. During the fermentation processes of lactic acid, sophorolipids and sodium gluconate, an online monitoring experimental platform based on non-contact near-infrared spectroscopy technology was established to conduct real-time monitoring of the fermentation process. A prediction model was established by combining partial least squares regression and internal cross-validation methods. In the sodium gluconate fermentation, the glucose concentration was optimized for control, and in the sophorolipid fermentation, the glucose and lipid concentrations were precisely regulated. As a result, the fermentation yields of sodium gluconate and sophorolipid were increased by 11.8% and 26.8%, respectively [102]. SSF and artificial intelligence, among other technologies, are driving the Baijiu industry towards revolutionary change.

**Figure 5:**
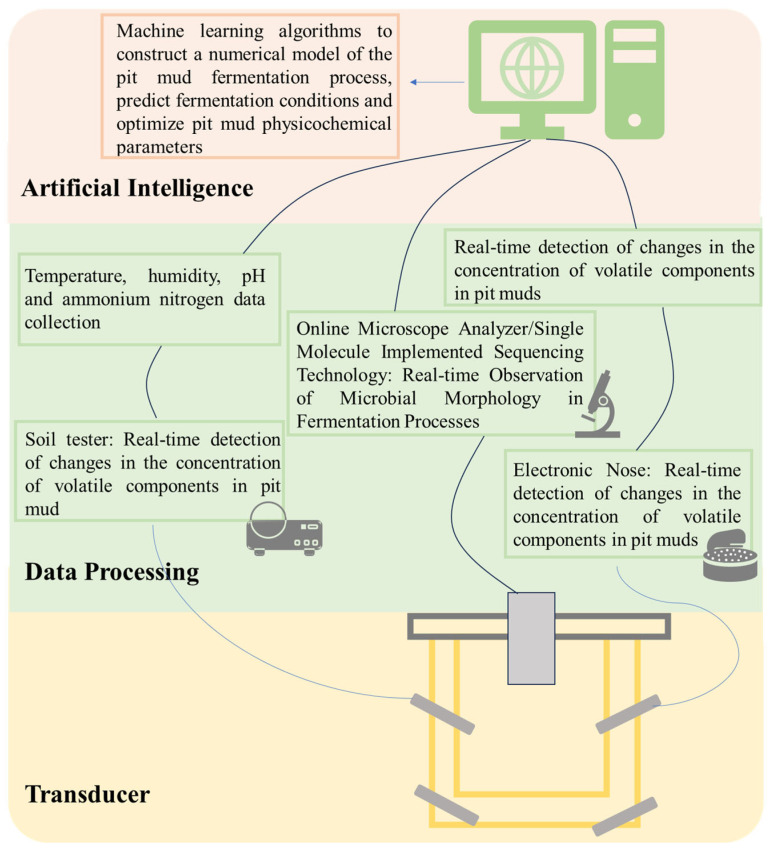
Trinity regulation strategy of pit mud quality.

During the quality control process of pit mud, it was found that the α and β diversity of prokaryotic communities exhibited significant changes along the pH gradient. Specifically, the new pit mud with lower pH had smaller species abundance, while the pit mud with higher pH had a stable biotic community structure, and the pit mud was of better quality [103]. In addition, different contents of humus were added to the culture process of pit mud for Baijiu brewing, and it was observed that the pit mud with high humus content could enhance the content of hexanoic acid in Baijiu while decreasing the lactic acid content. This resulted in a rapid improvement of both the original liquor quality and pit mud quality [96]. In the future, it is anticipated that a numerical model of the fermentation process can be developed during the regulation of pit mud quality. This will be achieved by connecting a soil tester to continuously collect physicochemical parameters of the pit mud, using high-throughput sequencing equipment to dynamically observe the microbial community, and employing electronic noses and other devices to monitor the volatile components of the pit mud. The model will be constructed by collecting extensive data and applying machine learning algorithms. The composition of microbial communities (e.g., dominant species and abundance changes) in pit mud can be predicted by environmental factors (e.g., pH, temperature, nutrients). Conversely, the environmental conditions can be inferred by microbial community characteristics. These models can predict potential issues during the fermentation process, such as reduced microbial activity or inadequate production of flavor compounds, and implement interventions proactively. For instance, adjustments to physicochemical factors, including pH, ammonium nitrogen, and humic substances, can alter the composition of the pit mud microbial community, thereby improving the quality of the pit mud [79] (as shown in Figure 5). The essence of these methods lies in employing artificial intelligence to tackle the complexity of the microbiome, extract rules from the data, and assist in solving the problems of “structure-function-environment” correlation analysis and optimization regulation of pit mud microorganisms. Intelligent control systems, when combined with physicochemical properties, demonstrate significant potential for regulating pit mud quality. However, they still encounter several challenges. The fermentation process of pit mud involves complex microbial interactions, the mechanisms of which remain incompletely elucidated. This lack of understanding presents a challenge for intelligent control. In the future, with the continuous advancement of technology, the application of intelligent control systems in pit mud fermentation will become more extensive and profound. During the process of pit mud quality control, these systems can optimize the function of the microbial community by precisely regulating key physicochemical parameters, thereby achieving targeted improvements in pit mud quality.

In summary, on the basis of clarifying the mechanism of the influence of the composition and content of physicochemical factors in APM on the functional microorganisms, we can adjust the composition of the functional microorganisms, control the types and contents of physicochemical factors, and construct synthetic microbial communities. This approach aims to regulate the quality of the pit mud and apply it to brewing production to increase the output rate and enhance the quality of Baijiu, which is significant for the health and quality development of the Baijiu industry.

## 6. Conclusions and Outlook

As the core fermentation reactor of SFB, the pit mud is still a significant bottleneck for quality improvement. The pit mud affects the flavor of SFB through the volatiles produced by pit mud microorganisms, and the succession and metabolism of these microorganisms are driven by the physicochemical properties of the pit mud. Therefore, a three-dimensional evaluation system (volatiles, microbial community and physicochemical properties) needs to be established for the control of the pit mud, and precise intervention can be achieved by analyzing the interactions among these three mechanisms. The environmental control strategy that alters the physicochemical properties of pit mud to enhance its functional microorganisms can compensate for the limitations of microbial control over pit mud quality. This approach aims to improve pit mud quality and develop high-quality APM. The crucial step to achieving this goal is identifying the driving factors of the microorganisms in pit mud through multi-omics linkage technology. It is anticipated that future control can be achieved by precisely managing the physicochemical parameters of pit mud, such as pH and ammonium nitrogen. The environmental control strategy based on changing the physicochemical properties of pit mud to enrich the functional microorganisms in pit mud can make up for the shortcomings of microbial control of pit mud quality and achieve the purpose of improving the quality of pit mud and developing high-quality artificial pit mud. The crucial step to achieving this goal is to identify the driving factors of the microorganisms in pit mud by using the multi-omics linkage technology. In the future, it is expected that the quality of pit mud can be accurately controlled by specifically regulating physicochemical parameters (such as pH value, ammonium nitrogen content, etc.), which can then be applied to the brewing of SFB to enhance the output rate and improve the quality of Baijiu.

## Figures and Tables

**Table 1:** Main microorganisms in pit mud.

Region	Brand	Main Microorganisms	References
Sichuan	Luzhou Laojiao	Bacteria: *Caproiciproducens*, *Lactobacillus*, *Petrimonas*, *Clostridium*, *Caldicoprobacter*, *Syntrophomonas*, Archaea: *Methanoculleus*, *Methanobrevibacter*, *Methanosarcina* Fungi: *Aspergillus*, *Monascus*, *Candida*, *Trichosporon*, *Byssochlamys*	[41,42,43]
	Wuliangye	Bacteria: *Lactobacillus*, *Caldicoprobacter*, *Clostridium*, *Thermoanaerobacterium*, *Caproicibacterium* Archaea: *Methanosarcina*, *Methanobacterium*, *Methanobrevibacter*, *Methanoculleus* Fungi: *Aspergillus*, *Thermoascus*, *Candida*, *Cryptococcus*, *Aminobacterium*	[44]
	Jiannanchun	Bacteria: *Lactobacillus*, *Bacillus*, *Clostridium*, *Garcielia*, *Petrimonas*, *Sedimentibacter*, *Syntophomonas*, *Petrimonas*, *Ruminococcaceae* Archaea: *Methanoculleus*, *Methanobrevibacter*, *Methanosarcina*, *Methanobacterium*	[45]
	Tuopai Qujiu	Bacteria: *Lactobacillus*, *Syntrophaceticus*, *Aminobacterium*, *Proteiniphilum*, *Syntrophaceticus*, *Petrimonas*, *Sedimentibacter*, *Ruminococcus*, *Ba cius*, *Acidobacterior*, *Proteobacteri*, *Propionibacterium*, *Pediococcus*, *Enterococcus*, *Vagococcus*, *Fastidiosipila*, *Caldiocprobacter* Fungi: *Fusarium*, *Aspergilus*, *Cladosporium*, *Ascomycota*, *Mortierellomycota*	[46]
	Shuijingfang	Bacteria: *Caprolciproducens*, *Clostridium*, *Lactobacillus*, *Caioramator*, *Syntrophomonas* Archaea: *Methanobacterium*, *Methanocueus*, *Methanobrevibacter*, *Methanocorpuscuilum*, *Methanosarcina*, *Methanobacterium* Fungi: *Dipodascus*, *Aspergillus*, *Thermoascus*, *Issatchenkia*, *Pichia*	[47]
Jiangsu	Yanghe Daqu	Bacteria: *Bacilli*, *Coriobacteriia*, *Lutispora*, *Caldicoproacter*, *Marinilabiliaceae*, *Tissierella*	[48]
Anhui	Gujing Gong	Bacteria: *Caldicoprobacter*, *Ruminiclostridium*, *Hydrogenispora*, *Caloribacterium*, *Proteiniphilum*, *Fermentimonas* Archaea: *Methanoculeus*	[49,50]
Henan	Songhe Grain Liquid	Bacteria: *Lactobacilus*, *Clostridium*, *Aminobacterium*, *Petrimonas*, *Syntrophomonas* Fungi: *Penicillium*, *Byssonectria*, *Mortierella*, *Thermomyces*, *Aspergillus*, *Rhizomucor*	[51]
	Yangshao	Bacteria: *Lactobacillus*, *Clostridium*, *Bacillus*, *Caproiciproducens*, *Lentimicrobium* Archaea: *Methanosarcina*, *Methanobacterium* Fungi: *Thermomyces*, *Rhizopus*, *Aspergillus*, *Thermoascus*, *Cladosporium*, *Thermomyces*	[52,53]
Inner Mongolia	Hetao King	Bacteria: *Lactobacilus*, *Caproiciproducens*, *Bacillus*, *Sphin-gomonas*, *Weissella*	[54]

## Data Availability

The original contributions presented in the study are included in the article, further inquiries can be directed to the corresponding author.
